# Induction of action-at-a-distance mutagenesis by 8-oxo-7,8-dihydroguanine in DNA pol λ-knockdown cells

**DOI:** 10.1186/s41021-015-0015-7

**Published:** 2015-08-01

**Authors:** Hiroyuki Kamiya, Masahiro Kurokawa, Tetsuaki Makino, Miwako Kobayashi, Ichiro Matsuoka

**Affiliations:** Graduate School of Science and Engineering, Ehime University, 2-5 Bunkyo-cho, Matsuyama, 790-8577 Japan; Graduate School of Biomedical and Health Sciences, Hiroshima University, 1-2-3 Kasumi, Minami-ku, Hiroshima 734-8553 Japan; College of Pharmaceutical Sciences, Matsuyama University, 4-2 Bunkyo-cho, Matsuyama, 790-8578 Japan

**Keywords:** 8-Oxo-7,8-dihydroguanine, 8-Hydroxyguanine, DNA polymerase λ, Knockdown, Action-at-a-distance mutagenesis

## Abstract

**Introduction:**

In DNA, 8-oxo-7,8-dihydroguanine (G^O^, 8-hydroxyguanine) is one of the most pivotal oxidatively damaged bases and induces G:C → T:A transversion mutations. DNA polymerase λ preferentially inserts dCTP opposite G^O^*in vitro,* and this error-free bypass function is considered to be important after A base removal from G^O^:A pairs by the MUTYH DNA glycosylase. To examine the effects of reduced levels of DNA polymerase λ on the G^O^-induced mutations, the polymerase was knocked-down in human U2OS cells, and a shuttle plasmid DNA containing a G^O^:C pair at position 122 in the *supF* gene was transfected into the cells. The plasmid DNA replicated in the cells was introduced into an *Escherichia coli* indicator strain, to measure the *supF* mutant frequency.

**Results:**

The knockdown of DNA polymerase λ significantly enhanced the mutant frequency of the G^O^ plasmid DNA. Contrary to our expectations, the knockdown did not promote the targeted G:C → T:A transversion. Instead, substitution mutations at G:C sites other than position 122 were enhanced in the cells.

**Conclusions:**

These results suggested that the knockdown of DNA polymerase λ induced action-at-a-distance mutagenesis in human cells when the G^O^:C pair was present in the DNA.

## Introduction

Oxidation of DNA and its related compounds by reactive oxygen species (ROS) is considered to cause mutagenesis, carcinogenesis, aging, and neurodegeneration [1, 2]. ROS are produced by environmental mutagens and carcinogens, as well as endogenously by normal oxygen metabolism, and thus function as enemies from within. Various oxidatively damaged nucleobases are reportedly generated, and 8-oxo-7,8-dihydroguanine (G^O^, also known as 8-hydroxyguanine) is one of the major damaged bases produced by ROS [3–5]. The G^O^ bases are estimated to form in 100–500 residues in DNA per cell per day [6].

The coding potential of this oxidized G base is ambiguous, and both the A and C bases have the ability to form pairs with G^O^ [7–10]. This nature causes G^O^ to be highly mutagenic, and the damaged base induces G:C → T:A and A:T → C:G transversion mutations when it is present in the DNA and the nucleotide pool, respectively, in mammalian cells [11–17]. To suppress the mutations caused by G^O^, various DNA repair and nucleotide pool sanitization enzymes exist to remove the damaged base/nucleotide from the DNA and the nucleotide pool, respectively [18, 19].

When dATP is incorporated by DNA polymerases (pols) opposite G^O^ in the template DNA, the G^O^:A pair is formed. The mammalian base excision repair enzyme, MUTYH DNA glycosylase, recognizes the G^O^:A pair and removes the unmodified A base from the base pair [20–23]. After the removal of A, the resultant gap is filled by DNA pols. Two X-family DNA pols, DNA pols β and λ, reportedly perform this gap-filling and incorporate dCTP and dATP opposite G^O^ in the gap [24, 25]. DNA pol λ, one of the translesion synthesis (TLS) DNA pols, is considered to be accurate for the G^O^ bypass, and dCTP is preferentially incorporated opposite G^O^. Thus, this MUTYH and DNA pol λ pathway could prevent the G:C → T:A transversion mutations caused by the oxidized G base. We have examined the effects of knockdowns of various cellular proteins on G^O^-induced mutagenesis in human cells [17–19, 26–29]. In cells with knocked-down MUTYH, the G:C → T:A transversion mutation induced by a G^O^:C pair is highly promoted [18]. Therefore, it was expected that similar results would be observed in living human cells with reduced amounts of DNA pol λ.

In this study, double-stranded plasmid DNA containing the G^O^:C pair in the *supF* gene was transfected into human U2OS cells, in which DNA pol λ was knocked-down. The DNA pol λ knockdown expectedly increased the *supF* mutant frequency when the G^O^:C plasmid DNA was introduced. However, the knockdown had no influence on the G:C → T:A transversion frequency, but remarkably enhanced the base-substitution mutations at untargeted G:C sites. These results suggested that the reduction of DNA pol λ unexpectedly induces action-at-a-distance mutagenesis by G^O^:C in human cells.

## Materials and methods

### Materials

Oligodeoxyribonucleotides (ODNs) containing G^O^ and G (G^O^-122, 5′-dCGACTTCGAAGG^O^TTCGAATC-3′; G-122, 5′-dCGACTTCGAAGGTTCGAATCC-3′), chemically phosphorylated at their 5′-end on the support during synthesis, were purchased from Nihon BioService (Asaka, Japan) and were purified by HPLC, as described previously [18, 30]. Other ODNs were obtained from Hokkaido System Science (Sapporo, Japan) and Sigma Genosys Japan (Ishikari, Japan) in purified forms. siRNAs (“stealth RNAi”, Life Technologies, Carlsbad, California, USA) were designed according to the BLOCK-iT RNAi Designer software, on the supplier’s Web site. The following siRNAs were used: pol λ sense, 5′-AAUAGAAGCAUCCUGCUCUGCCUUG-3′; and pol λ antisense, 5′-CAAGGCAGAGCAGGAUGCUUCUAUU-3′. Stealth RNAi Negative Control Medium GC duplex (%GC 48, Life Technologies) was used as the negative control.

The *Escherichia coli* strain KS40/pOF105 was provided by Professor Tatsuo Nunoshiba of International Christian University, and was used as an indicator strain of the *supF* mutants [31].

### Construction of plasmid DNA containing G^O^ and G

The double-stranded G^O^ and control plasmid DNAs were constructed from the single-stranded forms of pZ189-107 K/402E, and the G^O^-122 and G-122 ODNs, respectively, as described [32, 33].

### Mutagenesis experiments

The siRNA was introduced into U2OS cells with cationic liposomes as described [18]. The plasmid DNAs containing a single G:C or G^O^:C pair at position 122 of the *supF* gene were transfected into the U2OS cells by lipofection, at 24 h after the siRNA treatment. The transfected cells were cultured for 48 h, and the replicated DNA was recovered from the cells. The DNA was then introduced into the indicator KS40/pOF105 *E. coli* strain by electroporation, after the unreplicated DNA was removed by *Dpn* I treatment [31]. The electroporation experiments were repeated several times in single transfection experiments, and the transfection into U2OS cells was independently conducted three (G:C) and four (G^O^:C) times. The *supF* mutant frequencies were calculated as the ratios of white and faint blue colonies resistant to nalidixic acid and streptomycin.

### Western blotting

The western blotting was performed essentially as described [29]. The cell extracts were prepared with radio immuno-precipitation assay (RIPA) buffer containing protease inhibitors and were fractionated on an SDS-polyacrylamide gel. After transfer, the PVDF membranes were blocked in 3 % nonfat milk and probed with a rabbit anti-DNA pol λ antibody (catalogue no. A301-640A, Bethyl Laboratories, Montgomery, Texas, USA) or rabbit anti-GAPDH antibody (catalogue no. G9545, Sigma-Aldrich, St. Louis, Missouri, USA) for 1 h in Tris-buffered saline (TBS), containing 0.1 % Tween 20 and 3 % nonfat milk, at 20 °C. After washes, the membranes were incubated with horseradish peroxidase-conjugated anti-rabbit IgG (catalogue no. 7074S, Cell Signaling Technology, Danvers, Massachusetts, USA) for 1 h in TBS containing 0.1 % Tween 20 and 3 % nonfat milk, at 20 °C. After washes, the proteins were then visualized using the Enhanced Chemiluminescence (ECL) System (GE Healthcare Bio-Sciences, Piscataway, New Jersey, USA) and detected with an LAS 3000 Luminescent Image Analyzer (Fujifilm, Tokyo, Japan).

### Statistical analysis

Statistical significance was examined by the Student’s *t*-test. Levels of *P* < 0.05 were considered to be significant.

## Results

### The DNA pol λ knockdown enhanced the mutation frequency of G^O^:C

**Fig. 1:**
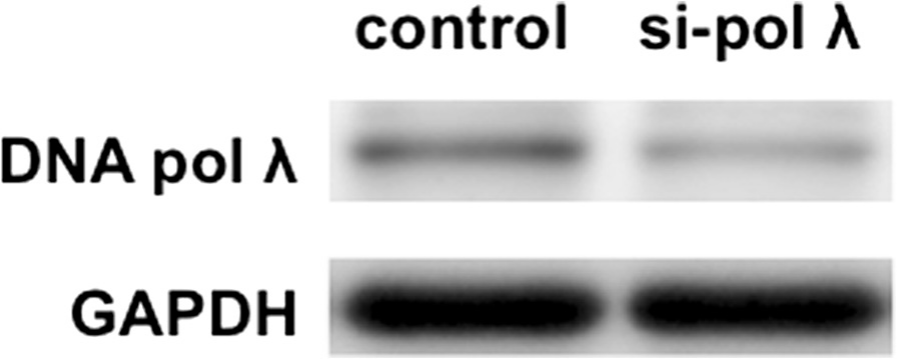
The levels of DNA pol λ expression in U2OS cells at 24 h after siRNA introduction, detected by western blot analysis

We examined the effects of the knockdown of DNA pol λ by siRNA on the mutations induced by G^O^. First, human U2OS cells were treated with the siRNA against this TLS DNA pol, and the knockdown was confirmed by western blotting using an anti-DNA pol λ antibody at 24 h post siRNA introduction (Fig. 1). The knockdown efficiency was calculated to be 34 % at 24 h after siRNA introduction.

**Fig. 2:**
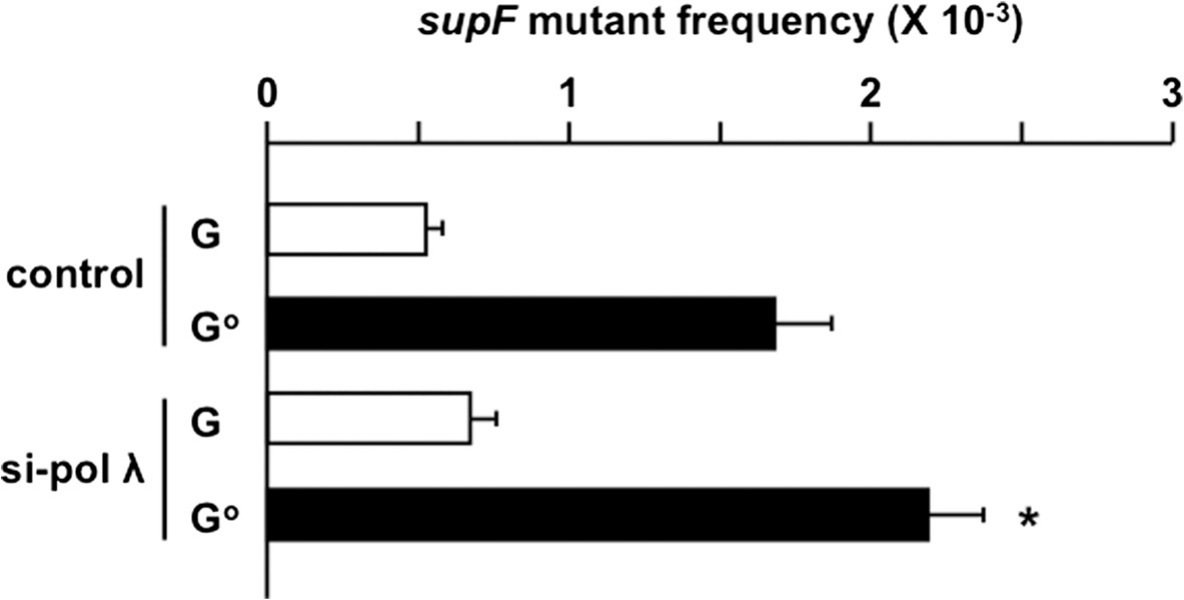
Effects of the DNA pol λ knockdown in U2OS cells on the mutant frequency induced by G^O^:C. Open columns, control plasmid containing G:C at position 122; closed columns, plasmid containing G^O^:C at position 122. Transfection experiments were performed three (G:C) and four (G^O^:C) times. Data are expressed as the means + standard errors. *P < 0.05 vs. control siRNA

Next, we transfected the plasmid DNAs containing a single G:C or G^O^:C pair at position 122 of the *supF* gene into the U2OS cells, after the siRNA treatment. The *supF* mutant frequencies were similar when the G:C plasmid was introduced into the cells treated with the siRNA against DNA pol λ and the control siRNA (~0.6 × 10^−3^, Fig. 2, open columns), indicating that the DNA pol λ reduction did not affect the background mutant frequency. The *supF* mutant frequency was 1.7 × 10^−3^ when the plasmid DNA with G^O^:C was introduced into the control siRNA-treated U2OS cells (Fig. 2). The mutant frequency was 2.2 × 10^−3^, representing a 1.3-fold (or 0.5 × 10^−3^) increase, when DNA pol λ was knocked-down (Fig. 2). These results suggested that DNA pol λ suppresses the mutagenesis caused by G^O^.

### The DNA pol λ knockdown induced action-at-a-distance mutations

DNA pol λ has been implicated as one of the DNA pols that fill the gap in an error-free manner, after MUTYH removes adenine opposite G^O^. Thus, we expected that the enhanced mutant frequency in the knockdown cells was due to increased G:C → T:A mutations at the target site, as MUTYH was knocked-down [18]. Therefore, we sequenced the *supF* genes from the colonies on the selection agar plates. Table 1 shows all of the mutations detected in the colonies obtained in this study. We analyzed 68 and 74 colonies derived from the G^O^:C plasmid DNA in the control and knockdown experiments, respectively. The spectra of the mutations found in the *supF* gene are summarized in Table 2.

**Table 1:** Mutations detected in the *supF* gene^a^

G : C			
control		si-pol λ	
74A- > G	1	91G- > A	1
95C- > G	2	91G- > C	1
98A- > G	1	95C- > G	1
101C- > A	1	96 T- > G	1
117C- > T	3	101C- > T	1
120A- > G	1	101C- > A	2
121G- > T	1	108C- > T	1
130C- > T	1	117C- > T	1
large deletion	9	118G- > A	2
		119A- > Δ	1
		125C- > T	1
		126G- > T	1
		large insertion	1
		large deletion	4
total colonies analyzed	20	total colonies analyzed	19
G^O^ : C			
control		si-pol λ	
27G- > A	1	61G- > A	2
61G- > A, 95C- > G, 111C- > T, 130C- > A, 153C- > T	1	61G- > A, 130C- > A	2
67G- > A	2	61G- > A, 153C- > T	1
72C- > G	1	71C- > G	3
85G- > T	3	86G- > A	1
91G- > C	1	86G- > A, 112G- > A, 126G- > C	2
95C- > T, 122G- > T, 125C- > G	1	91G- > A, 122G- > T	1
95C- > G	1	91G- > C	2
101C- > G	1	95C- > G	2
107 T- > G, 122G- > T	1	95C- > G, 111C- > T	1
112G- > T	1	101C- > T, 122G- > T	1
118G- > C	4	111C- > T, 153C- > T	2
118G- > C, 123 T- > G	1	118G- > A	2
121G- > T	5	118G- > C	5
122G- > T	36	121G- > A	3
123 T- > G	2	121G- > T	2
130C- > T	2	122G- > A	3
large deletion	4	122G- > T	29
		122G- > C	2
		126G- > C	2
		130C- > A	1
		large deletion	5
total colonies analyzed	68	total colonies analyzed	74

The numbers of colonies are shown on the right side

^a^Mutations detected in single colonies are represented. The sequence of the upper strand is shown

**Table 2:** Spectra of mutations detected in the *supF* gene

	G : C		G^O^ : C	
	Control	si-pol λ	Control	si-pol λ
mutations at position 122				
G : C - > A : T	0 (0)	0 (0)	0 (0)	3 (4)
G : C - > T : A	0 (0)	0 (0)	38 (56)	31 (42)
G : C - > C : G	0 (0)	0 (0)	0 (0)	2 (3)
mutations at other positions				
transition				
A : T - > G : C	3 (15)	0 (0)	0 (0)	0 (0)
G : C - > A : T	4 (20)	7 (37)	9 (13)	23 (31)
transversion				
A : T - > T : A	0 (0)	0 (0)	0 (0)	0 (0)
A : T - > C : G	0 (0)	1 (5)	4 (6)	0 (0)
G : C - > T : A	2 (10)	3 (16)	10 (15)	5 (7)
G : C - > C : G	2 (10)	2 (11)	11 (16)	17 (23)
small insertion (1–2 bp)	0 (0)	0 (0)	0 (0)	0 (0)
large insertion (>2 bp)	0 (0)	1 (5)	0 (0)	0 (0)
small deletion (1–2 bp)	0 (0)	1 (5)	0 (0)	0 (0)
large deletion (>2 bp)	9 (45)	4 (21)	4 (6)	5 (7)
others	0 (0)	0 (0)	0 (0)	0 (0)
total mutations	20	19	76	86
total colonies analyzed	20 (100)	19 (100)	68 (100)	74 (100)

All data are represented as cases found (%)

As observed in previous studies, the G:C → T:A transversion at position 122 (the modified site) was most frequently found in the colonies obtained from cells treated with the control siRNA and the G^O^:C plasmid [18, 26, 27]. The ratio of the targeted G:C → T:A transversion was high, at 56 % (Table 2). In contrast to our initial expectation, however, the ratio of the targeted G:C → T:A mutation decreased to 42 % for the DNA pol λ-knockdown cells (Table 2), although the total mutant frequency was 0.5 × 10^−3^ higher in the knockdown cells than in the control cells. We then calculated the frequencies of the targeted G:C → T:A transversion, by multiplying the total *supF* mutant frequencies by the percentages of the targeted G:C → T:A mutation. The calculated G:C → T:A frequencies were 1.0 and 0.9 × 10^−3^ in the control and DNA pol λ experiments, respectively, indicating that the DNA pol λ knockdown did not promote the targeted mutation. Thus, the observed 0.5 × 10^−3^ higher total mutant frequency was due to other mutations.

We noticed that substitution mutations at G:C sites (G:C → A:T, G:C → T:A, and G:C → C:G mutations) other than position 122 were frequently found (Table 2). The ratios of the untargeted substitutions at G:C sites were 44 % (30 mutations for the 68 colonies analyzed) and 61 % (45 mutations for the 74 colonies analyzed) in the control and DNA pol λ-knockdown cells, respectively. In the knockdown cells, the untargeted substitution mutations at G:C pairs were more abundant than the targeted G:C → T:A transversion. These results were similar to those observed when the Werner syndrome protein (WRN) was knocked-down [29]. We multiplied the total *supF* mutant frequencies by the percentages of the untargeted substitutions at G:C sites for each transfection experiment. The calculated frequencies were 0.6 (±0.2) and 1.4 (±0.3) × 10^−3^ in the control and knockdown cells, respectively (standard errors are shown in parentheses). Thus, the untargeted substitutions were likely to occur by the DNA pol λ knockdown, although these values were statistically insignificant (*P* = 0.10). We then examined the mutational patterns in the DNA strand containing the G^O^ base (the upper strand of the plasmid) when DNA pol λ was knocked-down. Statistical significance was observed, and the untargeted substitution frequencies at the G sites were 0.4 (±0.1) and 0.8 (±0.1) × 10^−3^ in the control and knockdown cells, respectively (*P* < 0.05). Based on these results, we concluded that substitution mutations at G (or G:C) sites were increased in the DNA pol λ-knockdown cells. Thus, the G^O^ base induced action-at-a-distance mutations when the level of DNA pol λ was reduced.

**Fig. 3:**
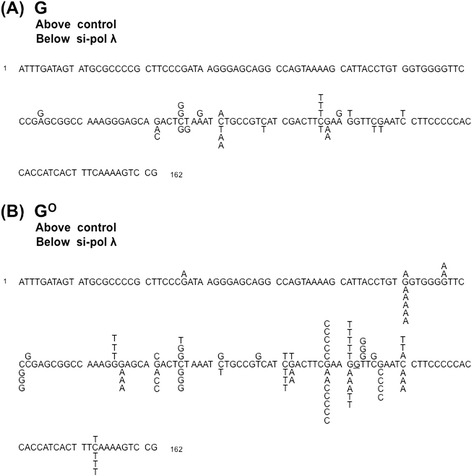
Overall distribution of the substitution mutations detected in the *supF* gene, based on the data shown in Table 1. The sequence of the upper strand of the plasmid is shown. The mutations obtained with the control and anti-pol λ siRNAs are shown above and below the sequence, respectively. **a** The control plasmid containing a G:C pair at position 122. **b** The plasmid containing a G^O^:C pair at position 122. The underlined G in panel B represents the 122nd position, and the targeted mutations are not depicted

The distributions of the mutations, except for those at the target position, are shown in Fig. 3b for the G^O^ experiments. The untargeted mutations were observed in the region from ~60 bp upstream to ~30 bp downstream of position 122, the G^O^ site.

## Discussion

The objective of this study was to examine the effects of DNA pol λ knockdown on the mutations induced by the G^O^ base in DNA. Since DNA pol λ is considered to function in an error-free manner when the gap is filled, after MUTYH removes adenine opposite G^O^, the reduction of the TLS DNA pol was expected to enhance the G:C → T:A transversion mutation. As shown in Fig. 2, the reduction in the protein level significantly increased the G^O^-induced mutations. Unexpectedly, however, sequence analyses indicated that the targeted G:C → T:A mutation was not increased (Table 2). Instead, substitution mutations at G:C sites other than the targeted position were frequently found. Thus, DNA pol λ may suppress action-at-a-distance mutations in living human cells.

We recently found action-at-a-distance mutations induced by G^O^ in cells with knocked-down WRN, although the molecular mechanism(s) of the mutations in the WRN-knockdown cells still remain unknown [29]. Interestingly, Kanagaraj et al. reported that DNA pol λ interacted with WRN [34]. They found that WRN physically bound to DNA pol λ and enhanced the gap-filling activity of the DNA pol. Moreover, the two proteins reportedly formed nuclear foci induced by oxidative stress, in a DNA replication- and MUTYH-dependent manner. Based on the report by Kanagaraj et al. and our previous and present studies, DNA pol λ and WRN could cooperatively prevent the action-at-a-distance mutations. Error-prone DNA pol(s) might be involved in DNA synthesis reactions in DNA regions near the G^O^ base upon DNA pol λ or WRN reduction. However, as discussed in our previous report [29], this hypothesis is apparently invalid, since mismatch formation by error-prone TLS DNA pols is more likely to occur at A:T sites rather than G:C sites [35–39].

We previously suggested some possible reasons for the action-at-a-distance mutations induced by G^O^ in the WRN-knockdown cells. The first possibility is the increased mismatch formation generated by the reduction of the 3′ → 5′ WRN exonuclease activity, plus its further inhibition in the presence of the G^O^ base [40–43]. Alternatively, the formation of G^O^ radicals and the following radical cation migration on DNA might cause the action-at-a-distance mutations, because of a prooxidant state in the WRN-reduced cells [44–52]. Although other possibilities are not completely excluded, these explanations are likely to be valid, based on the hypothesis that the inability to interact with WRN is important for the action-at-a-distance mutations in the DNA pol λ-knockdown cells. Since DNA pol λ interacts with WRN, the DNA pol λ knockdown might affect the intracellular fate of WRN, including its stability. The effects of DNA pol λ knockdown on the WRN protein are currently examined from various viewpoints in our laboratory.

Previously, Efrati et al. reported “action-at-a-distance mutagenesis” in *in vitro* DNA synthesis reactions conducted by DNA pol β when G^O^ was present in the template [53]. The authors of the paper observed misincorporation of deoxyribonucleotides opposite the 3′-base adjacent to the lesion. DNA pol β might act in the gap after MUTYH removes adenine opposite G^O^ in the DNA pol λ-knockdown cells more frequently than in the control cells. One may think that similar events contributed to the action-at-a-distance mutagenesis observed in the knockdown cells. However, no mutations were found at position 123, the 3′-flanking T base, in the knockdown cells (Fig. 3b, below the sequence). Thus, the misincorporation opposite the 3′-base adjacent to G^O^ observed in the *in vitro* DNA synthesis reactions seemed to play little, if any, roles under our experimental conditions.

The knockdown efficiency of DNA pol λ was calculated to be 34 % at 24 h after siRNA introduction, which was the time point when the plasmid DNAs were transfected (Fig. 1). Since the knockdown promoted the *supF* mutant frequency, the knockdown with this efficiency certainly affected mutagenesis by the G^O^ base (Fig. 2). However, the knockdown efficiency would decrease during 48 h between the transfection and the plasmid recovery. Thus, more drastic effects could be observed when the knockdown is more effective or when DNA pol λ-knockout cells are used.

We were able to detect the induction of the action-at-a-distance mutagenesis, since we employed the site-directed introduction of the single G^O^ base into the shuttle plasmid used for the transfection experiments. Thus, this work confirms that experiments using these types of plasmid DNAs are useful in mutagenesis studies [3].

Pande et al. examined the mutagenesis induced by G^O^ in DNA pol λ-knockdown human cells, using a single-stranded plasmid and ODN hybridization (followed by sequencing) [54]. In agreement with our present finding, no increase in the targeted G:C → T:A transversion was observed. In contrast, the authors did not mention the induction of action-at-a-distance mutations at G/C sites. This discrepancy could be due to the differences in the G^O^-DNAs used for the transfection experiments (single-stranded and double-stranded plasmid DNAs) and the mutant selection methods (ODN hybridization and phenotypic selection). Thus, our results shown in this study constitute the first evidence that the reduction of DNA pol λ resulted in the untargeted mutations by G^O^ in human cells.

## Conclusions

In conclusion, action-at-a-distance mutations at G:C (or G) sites were induced by G^O^ in the DNA pol λ-knockdown cells. Further studies are necessary to elucidate the detailed mechanism(s) of the untargeted mutagenesis, and are currently in progress.

## Abbreviations

G^O^: 8-oxo-7,8-dihydroguanine
ODN: Oligodeoxyribonucleotide
pol: Polymerase
ROS: Reactive oxygen species
TLS: Translesion synthesis
